# Routine Outcome Monitoring and Clinical Feedback in Psychotherapy: Recent Advances and Future Directions

**DOI:** 10.1007/s10488-024-01351-9

**Published:** 2024-02-08

**Authors:** Andrew A. McAleavey, Kim de Jong, Helene A. Nissen-Lie, James F. Boswell, Christian Moltu, Wolfgang Lutz

**Affiliations:** 1 Helse Førde Hospital Trust, Svanehaugvegen 2, Førde, 6812 Norway; 2 Department of Health and Caring Science, Western Norway University of Applied Science, Førde, Norway; 3 Department of Psychiatry, Weill Cornell Medical Center, New York, NY USA; 4 Institute of Psychology, Clinical Psychology Unit, Leiden University, Leiden, The Netherlands; 5 Institute of Psychology, University of Oslo, Oslo, Norway; 6 Department of Psychology, University at Albany, State University of New York, Albany, NY USA; 7 Department of Psychology, University of Trier, Trier, Germany

**Keywords:** Routine outcome measurement, Psychotherapy, Clinical feedback, Measurement-based care

## Abstract

In the past decade, there has been an increase in research related to the routine collection and active use of standardized patient data in psychotherapy. Research has increasingly focused on personalization of care to patients, clinical skills and interventions that modulate treatment outcomes, and implementation strategies, all of which appear to enhance the beneficial effects of ROM and feedback. In this article, we summarize trends and recent advances in the research on this topic and identify several essential directions for the field in the short to medium term. We anticipate a broadening of research from the focus on average effects to greater specificity around what kinds of feedback, provided at what time, to which individuals, in what settings, are most beneficial. We also propose that the field needs to focus on issues of health equity, ensuring that ROM can be a vehicle for increased wellbeing for those who need it most. The complexity of mental healthcare systems means that there may be multiple viable measurement solutions with varying costs and benefits to diverse stakeholders in different treatment contexts, and research is needed to identify the most influential components in each of these contexts.

As psychotherapy has developed from a primarily theoretically defined clinical intervention to a more evidence-based one in the past 100 years, measuring patient outcomes has become increasingly important. While psychological measurements were developed and implemented in some branches of psychotherapy as early as the middle of the 20th century (e.g., Knight, 1941), and using standardized self-report questionnaires to track patients’ progress has been a longstanding recommendation for therapists (e.g., Beck et al., 1961), this was far from common (De Jong et al., 2023). Today, the regular collection of standardized patient data is considered an evidence-based adjunct across a range of clinical settings and treatment types (Boswell et al., 2022; Coalition for the Advancement and Application of Psychological Science, 2018). Often, patient-rated data is collected as part of routine practice and reviewed by psychotherapists to better address the patient’s specific needs. This process is variously called routine outcome monitoring (ROM), clinical feedback (CF), measurement-based care (MBC), feedback-informed treatment (FIT), and other terms. In this manuscript, we are using the term ROM and feedback to include all of these. Some health authorities have even mandated that mental health care providers use some degree of measurement or screening routinely (Joint Commission, 2018), based on evidence that it improves outcomes on average, decreases the frequencies of negative outcomes among those most at risk for treatment failure, and/or improves cost-effectiveness (Barkham et al., 2023; De Jong et al., 2021).

The goal of this paper is to summarize recent progress towards understanding the uses of ROM and feedback, and identify key future directions for research and clinical practice. We will start with a very brief summary of a broad current understanding of ROM and feedback in psychotherapy. Following this, we will explore a small number of topics that have been the subject of recent research. We will conclude with discussion of the future: What questions regarding ROM and feedback require attention from clinical scientists, in order to improve the care for patients in psychotherapy?

## What We Already Know about ROM And Feedback – We Think

Previous reviews of ROM and feedback in psychotherapy have illustrated a few fairly consistent findings. We think we can justify claims that ROM and feedback improves patient outcomes; that this benefit to patients is heterogeneous, with some patients benefiting greatly and others very little; that larger effects are observed with more complete implementation strategies; that existing ROM and feedback systems are generally cost-effective from a system-of-care perspective, and that measures themselves have an impact on patients. These claims, elaborated below, are provided with substantial caveats: The findings summarized here often derive from relatively weakly powered and low-quality studies, and there are likely areas in which the available evidence is quite weak. There is some reason for skepticism around each of these points, and future findings could modify these statements considerably.

First, it seems that ROM and feedback improves patient outcomes on average by a small effect size, but likely in heterogeneous ways across several moderators. While a Cochrane review found insufficient evidence to support the use of ROM and feedback (Kendrick et al., 2016), other reviews and meta-analyses have concluded that these tools decrease negative outcomes such as dropout and symptomatic deterioration when used as feedback tools. A meta-analysis by De Jong and colleagues (De Jong et al., 2021) found small but significant effects of ROM and feedback on symptom reduction and dropout. They also identified several important moderators of these effects, including specific instrument used for feedback, frequency of use, treatment intensity, and which measure was used as the marker of outcome. Thus, while using ROM and feedback is not a universally positive intervention, it seems to generate improvement on the margin, with stronger (and weaker) effects in several known circumstances. Many of the most common explanatory theories for this impact include the active and explicit use of ROM by therapists to flag cases with limited improvement and then re-evaluate and/or change the treatment plan. Because this means ROM and feedback relies on clinical skill and is embedded in an existing psychotherapeutic treatment, it should not be surprising that positive effects do not occur universally: no psychological intervention is effective in all cases.

In part because the positive effects of ROM and feedback are dependent on several factors, a major focus of research has been the implementation of ROM and feedback: What are the major barriers to successful use of this intervention, and what facilitates its uptake? Simply giving therapists and patients questionnaires does not lead to improved psychotherapy. Instead, attention to training, ensuring the measures are actually used, and providing ample support to staff are among the methods that appear to increase use of ROM and feedback (Lewis et al., 2019). There are substantial differences between therapists which impact the potency of feedback interventions, and suggests that different implementation methods will work with different therapists (Janse et al., 2023). There is also some evidence that therapists increase their use of ROM and feedback with training (Brattland et al., 2018), and therapists’ use of these tools is predicted by their beliefs about the measurement’s practical benefits (Kwan et al., 2021) and validity of the feedback (De Jong et al., 2012). Across clinical treatment delivery centers, there are important differences in how ROM and feedback systems are implemented (Bickman et al., 2016). Some treatment centers improve noticeably after implementing ROM and feedback, but not all (Bickman et al., 2016; Bovendeerd et al., 2021). This illustrates the importance of implementation strategies, training, and support in the effective use of ROM and feedback, and suggests that greater training, education, and support across treatment centers likely will lead to more noticeable improvements. ROM and feedback includes a substantial obligation for clinic administrators to provide support to therapists, it is not simply a matter of providing questionnaires in a waiting room.

Even if ROM and feedback tools are effective in improving the outcomes of psychotherapy, it is important to examine how much benefit they provide relative to the costs they incur. Cost analyses depend on many factors, including the incremental cost of each measurement, the baseline cost of care (which varies by setting, treatment intensity, and payment structure), desired outcomes, and other system-specific issues. One key driver for costs in mental healthcare is treatment length, often represented by number of treatment sessions in outpatient psychotherapy, since the costs of psychotherapy are closely related to therapists’ hourly pay. There is some evidence that ROM and feedback reduces treatment length, which means it likely reduces systemic costs. Using a cohort design, Delgadillo and colleagues (2017) demonstrated that psychotherapy with feedback from the GAD-7 and PHQ-9 resulted in similar outcomes to psychotherapy without that feedback, but with a shorter average treatment length. This suggests a substantial cost-effectiveness benefit of psychotherapy with ROM and feedback systems. In a later randomized trial, Delgadillo and colleagues (Delgadillo et al., 2021) found that using feedback led to improved outcomes at a modest cost per patient. If ROM and feedback reduces costs of care, even without improving outcomes, there is an argument to be made that not using it is a waste of time and money. The major limitation of this cost effectiveness research is that it has only included a handful of studies, in only a limited number of settings, so greater analysis is required before universal recommendations are made.

In addition to quantitative research, there has also been an increase in qualitative studies of ROM and feedback (Låver et al., 2023). From this research, it is clear that the simple act of completing a measure has an impact on patients and adding ROM and feedback to a psychotherapy is inherently impactful to care. Patient experience of measurement seems to be a key variable in some cases. These impacts are diverse, but include causing patients to reflect on their own functioning and/or becoming suspicious about how their providers (or others) will use their answers (Solstad et al., 2019). Moreover, the clinical context of measurement influences patient responses, such that they should not be taken as unbiased symptom ratings (Truijens et al., 2021). Patients sometimes describe motivated responding, in which they alter their response to fit a perceived goal, such as communicating specific information to guide treatment or managing others’ impressions of themselves (Hovland et al., 2020). These and other findings suggest new ways of understanding the impact of ROM and feedback, and might help further explain the heterogeneity of effects.

In summary, there is support for ROM and feedback as an effective and potentially cost-effective adjunct to individual psychotherapies. However, while some studies have shown strong support, not every study of ROM and feedback has demonstrated benefit. These differences in effect may be due to several factors such as implementation strategy, measurement fit with the patient, therapist, and clinical setting, and the patients’ experience of measurement in the context of their care. The field needs clarity and direction: How can research on ROM and feedback address these varied influences, and how can we use empirical knowledge to better achieve clinical gains? In the next sections, we summarize several key areas of recent research in ROM and feedback, which have revealed important new directions for the field. Following this, we look forward and consider what the next decade(s) of research will entail.

## Recent Advances

### Personalized and Dynamic Feedback Systems

Psychotherapy has always been a personalized treatment: psychotherapists must adjust general plans, strategies, and techniques to their patients’ specific circumstances. Appropriate deviations in treatment plan based on patient needs has been a longstanding hallmark of effective therapy (Stiles & Horvath, 2017). ROM and feedback provide a more formal and measurable approach to personalize treatment, relying less on clinical judgement. While the formative work on ROM and feedback used standardized measures only at convenient times and provided limited flexibility, more recent work with ROM and feedback has pushed the boundaries of how person-specific feedback can be. There are three groups of advances emerging in research now: (1) Personalizing treatment using routinely-collected pre-treatment data; (2) Questionnaires that are at least partially person-specific, and (3) Statistical methods that are dynamically responsive to each patient. The foundation for all of these advances remains structured data provided routinely by patients to therapists.

The first trend, personalized treatment plans, has emerged in research focused on determining the optimal way to tailor treatment plans or strategies to suit specific patient characteristics and needs. This research is moving away from the patient uniformity myth (Kiesler, 1966), in which everyone with the same diagnosis should receive the same type of treatment, and instead aims to personalize psychological therapy. This approach shares similarities with the broader discussion around precision medicine, in that it is an attempt to match a treatment to a patient (e.g., Chekroud et al., 2021; Cohen et al., 2021; Delgadillo & Lutz, 2020). Multiple examples of routine data-driven treatment assignment have been tested. One relatively advanced approach was recently tested in a multisite, cluster-randomized controlled trial of 950 patients in the Improving Access to Psychological Therapies (IAPT) program (Delgadillo et al., 2022). Using machine learning prediction models based on patient-reported variables, the researchers identified cases who were considered “complex” and therefore might benefit from higher-intensity treatment. The results showed that using this method was more effective and cost-effective than the standard stepped care. Because the standard stepped care was itself based on a limited set of patient-reported data with a simpler decision rule, this demonstrates that accounting for more routinely-available patient information can be useful in treatment selection. Beyond treatment selection, several treatments have become modular, meaning that some of the content and skills can be provided earlier or later in treatment than others (Barlow et al., 2020). An important component of this work has been the use of intensive longitudinal data in a pretreatment phase to identify patient-specific key symptoms. Fisher (2015) demonstrated that patients with similar diagnoses can have markedly different patterns of change on depressive symptoms over time. This idea has inspired several efforts to develop optimal module ordering as a personalized treatment approach (Rubel et al., 2018), with some initial promising results (Sauer-Zavala et al., 2019; Southward et al., 2023). It should be noted that there are still substantial questions regarding how much benefit and how consistent any benefits of these highly personalized approaches will be in practice. One challenge is that there is a great deal of flexibility in applying such methods, leading different researchers to different conclusions even with the same data (Bastiaansen et al., 2020). Additionally, the reliability of these models and their consistency over time may be limited except with a large number of observations per person (Bringmann, 2021). Nevertheless, these methods are highly promising and future developments may alleviate some of these issues.

The second trend, more personalized questionnaires, has come about in multiple ways. One approach with long history has been to have patients identify for themselves one or several key issues to track during treatment. This approach may have been pioneered by Shapiro (1961). Given an idiographic item, quantitative tracking similar to that of standardized questionnaires can be possible, and even appears to have similar psychometric properties (Elliott et al., 2016). These fully idiographic measures avoid some of the criticisms of structured questionnaires by ensuring that individual patients track meaningful constructs (Sales et al., 2022). Studies using these forms indicate some clear benefit: many patients identify topics to measure and track that are not covered in standardized questionnaires (Sales et al., 2018). A second way to personalize questionnaires is exemplified by the multidimensional, partially computer-adaptive Norse Feedback (NF) system (McAleavey et al., 2021; Nordberg et al., 2021). The NF consists of 18–22 brief scales that are not provided to each patient at every visit, but rather algorithmically determined based on patient answers. That is, some patients will answer questions related to sad affect, while others will answer more questions about eating disorders. This type of system affords a different balance of structure and personalization in ROM and feedback.

The third trend in achieving precision in psychological therapy involves methods to improve treatments for patients at risk of treatment failure (e.g., Barkham et al., 2023; Lutz et al., 2021). The foundation of this practice is continuous data collection to create evidence-based decision-making tools that guide individual treatment decisions before and during treatment, as well as decisions on the level of clinical services. One of the main benefits of ROM and feedback has historically been to identify patients whose scores put them at risk for treatment failure, using relatively straightforward methods. Recently, dynamic statistical models – methods that respond to data as it comes in – have been applied to discover key features that can be used by the therapist to adjust treatment to a specific person at a specific point in treatment. Though many of these methods are complex and computationally sophisticated, this idea has been fruitfully explored for several decades: Molenaar (1987), for instance, illustrated in a single subject the changing relationships between therapist actions and the patient’s complaints over the course of treatment. For that patient, different clinical strategies (e.g., increasing counseling/modeling behaviors, or changing the therapist’s interactive style) had varying and even opposite effects depending on the stage of treatment. This pioneering work was accomplished with linear models and relatively simple estimation techniques (by modern standards). Advances in statistical machine learning methods, as well as new digital assessments and feedback tools, enable even more sophisticated development and implementation of these decision-making tools (e.g., Cohen et al., 2021; Delgadillo & Lutz, 2020; Lutz et al., 2022).

The Trier Treatment Navigator (TTN) is an example of a system that supports clinical decision making at the beginning and during therapy and has been tested prospectively (Lutz et al., 2019, 2022a). At the beginning of therapy, a machine learning algorithm is used to tailor treatment strategies to the specific needs of the patient (problem-solving vs. motivation-oriented) and to calculate individual dropout risks. However, even with optimal tailoring at the start of treatment, successful outcomes are not guaranteed. Therefore, the TTN further supports ongoing treatment decisions by providing feedback on patient progress, clinical material, and support tools for patients at risk of treatment failure. The TTN allows therapists to evaluate treatment progress and provides a dynamic threshold to indicate whether changes are as predicted or whether they indicate an increased risk for negative treatment outcomes. That is, the threshold for considering treatment to be a failure not only changes for each patient, but changes over the course of therapy for each patient.

A randomized controlled trial of 538 patients was conducted to prospectively evaluate the TTN. Results showed that if therapists followed the recommended treatment strategy for the first ten sessions, better outcomes resulted by an effect size of about 0.3. In addition, therapist symptom awareness, attitude toward and confidence in feedback were significant predictors of outcome, whereas therapist perception of feedback usefulness was a significant moderator of the feedback-outcome and not-on-track-outcome associations (Lutz et al., 2022). These findings highlight the potential of such decision-support systems and the importance of implementing them well in practice.

In another example, Bone et al. (2021) used a dynamically-updated alarm signal for each patient. While their results were retrospective, when they applied their model to a separate held-out dataset (which had not been used to generate the model), they found that using the dynamic model improved early prediction of treatment failure, suggesting it would be preferable to use this method over the one-size-fits-all approach. This suggests that data-driven decisions at baseline and throughout treatment can improve the management of mental health symptoms.

Despite some recent studies validating treatment selection models, further validation studies, including prospective and implementation studies, are needed to ensure the accuracy and effectiveness of these models (Cohen et al., 2021). Independent holdout data (as described above), data from other trials (cross-trial validation), and prospective validation/implementation studies in actual clinical trials have been used to validate treatment selection models (e.g., Delgadillo et al., 2022; Lutz et al., 2022; Schwartz et al., 2021). However, more studies in this direction are needed. In addition to further validation studies, future developments such as more intensive interventions multiple times per day, digital phenotyping of stress markers, or data from video analysis of emotions and clinical processes may further enhance treatment prediction and monitoring systems (Lutz et al., 2022).

### Patient-therapist Matching

Over time, as ROM data are collected to support feedback-informed treatment in increasing and varied contexts, a vast amount of patient-level outcome data accumulates. As patient-level outcome data accumulate, so does therapist-level outcome data. ROM data can provide information on individual therapist outcomes by estimating the average outcome (or amount of change experienced) among patients in a given therapist’s caseload. In fact, Howard et al. (1996) explicitly stated that treatment response monitoring can facilitate provider-level comparisons (after case-mix adjustments are applied). Research conducted in both controlled and naturalistic settings has demonstrated average caseload outcome differences among therapists that are estimated to account for approximately 5% of the variance (labeled therapist effects; see Johns et al., 2019).

Recent advances have moved beyond the basic statistical observation that some therapists evidence better patient outcomes than others to address the nuances of observed differences and how therapist-level outcomes data might be harnessed to improve patient outcomes and overall quality of care. One prominent focus has been the implications of therapist-level practice-based evidence for referring or assigning patients to therapists (Boswell et al., 2017; Delgadillo et al., 2020). Although exceptions are sure to exist, the therapist selection, referral, and assignment process is often unsystematic (at least in the United States) and typically based on convenience and therapist-self-identified expertise (Boswell et al., 2021). As a given patient’s likelihood of experiencing a treatment benefit appears to be in part a function of who their therapist is, it is unfortunately left somewhat up to chance that a patient will end up working with an empirically well-suited therapist. This concern is further exemplified by research findings that therapist tend to overestimate their effectiveness (Walfish et al., 2012).

When ROM tools generate unidimensional data or global scores (e.g., global distress or impairment), a basic level of therapist differentiation and empirical therapist selection can be implemented. Specifically, new patients can be steered to therapists who have evidenced relatively superior outcomes, and away from therapists who have evidenced relatively poorer outcomes (Imel et al., 2015). Other research using ROM data has demonstrated that the therapist matters more for certain types of patients. In particular, it appears to be more important for patients with higher clinical severity to be matched with better performing therapists; conversely, the therapist appears to matter less in predicting outcomes for patients who enter therapy with mild severity or impairment (Johns et al., 2019).

Additional research has been conducted with multidimensional ROM tools, posing the question of whether or not therapist effectiveness is better perceived as a global or a multidimensional construct. The potential variability in therapist effects has been explored across different patient outcome/presenting problem domains and outcome monitoring systems. For example, Nissen-Lie et al. (2016) found support for a global therapist effectiveness construct, rather than evidence supporting the idea that therapists have distinct effects on different symptom types (that is, they found that effective therapists are generally highly effective across all domains of patient complains, not that therapists are often highly effective at treating depression while also highly ineffective at treating, say, problems in social relationships). In contrast, other research involving a different ROM system has observed that therapist effectiveness can vary based on the patient outcome domain. For example, the Treatment Outcome Package (TOP; Kraus et al., 2005) consists of 58 items assessing 12 symptom and functional domains. Therapist effects have been observed on each of the TOP outcome domains (Kraus et al., 2011), including after case-mix adjustments (Kraus et al., 2016). In addition, within their own caseloads, therapists appear to demonstrate a relative pattern of effectiveness or ineffectiveness depending on the outcome domain. A given therapist may be particularly effective at producing positive changes in their patients’ depression and anxiety, yet this same therapist may be particularly ineffective at producing changes in their patients’ substance misuse or sleep problems. Other therapists may possess the reverse pattern of relative strength and weakness.

Therapist outcome patterns also appear to be stable (Kraus et al., 2016; Wampold & Brown, 2005). If therapist outcome track records are stable and certain therapists have been empirically demonstrated to be more suitable for certain patients (and less suitable for others), then there is potential for a more personalized patient-therapist matching approach based on ROM. Outcome-domain focused patient-therapist matching was tested in a double-masked randomized controlled trial (RCT). Routinely presenting patients were randomized either to a case assignment as usual (CAU) control group or to a personalized Match experimental group based on therapists’ multidimensional track records as determined by historical patient pre-post TOP data (Constantino et al., 2021). Psychotherapy in both conditions was then delivered naturalistically. Results demonstrated that Match vs. CAU patients reported significantly greater reductions in general symptomatic/functional impairment across 16 weeks of care (*d* = 0.75). Such tailored patient-therapist matching represents an advancement in the personalization of psychotherapy. As a replication and extension of previous findings, the benefit of the Match effect was found to be more pronounced with patients who entered treatment with higher levels of clinical severity and who identified as a racial/ethnic minority individual (Boswell et al., 2022).

Making use of this patient-therapist matching effect in practice requires data from ROM, because therapists tend not to be very accurate at identifying their own relative strengths and weaknesses. Using multilevel models to test whether therapists’ problem-specific perceptions predicted global between-therapist performance differences, Constantino et al. (2023), found that therapists who consistently overestimated their problem-specific effectiveness had patients who reported worse global outcomes compared to patients whose therapists more accurately estimated their effectiveness. Based on this finding, the authors conclude that “therapist humility may differentiate the most from least globally effective therapists, and this virtue should be cultivated in clinical trainings” (p. 474). In addition, Delgadillo et al. (2020) recently examined a large sample of routinely presenting patients with ROM and case-mix data and found that specific subgroups of therapists were more or less able to help specific subgroups of patients (e.g., defined by initial severity or employment status).

The feasibility of implementing such outcome-based matching (based on presenting problem domain and/or other patient characteristics) in routine mental health care settings requires more attention. One of the authors is presently involved in a large-scale implementation project that aims to integrate ROM and outcome-based patient-therapist matching as part of routine clinical practice in a large mental health care system in the United States.

### Understanding ROM and Feedback as Integrated with Therapeutic Processes

Measuring symptoms and processes in psychotherapy was initially understood as relatively independent from the clinical processes that transpire in the therapy dyad. In recent years, however, this perspective has been challenged. While a thermometer will read, but not influence, a person’s temperature, inviting patients to rate their experiences during psychotherapy may, for example, direct attention to particular areas and away from others, influence perceptions, reinforce certain topics, and implicitly communicate what the provider finds relevant to the patient (Solstad et al., 2020, 2021b). These fundamental differences between physical and psychological measures have been increasingly explored, revealing reasons to consider measurement to be an active and essential part of the therapeutic process. As any active component, it can have both positive and negative influences.

On the negative side, some studies have found that ROM and feedback can limit, or at least fail to capture, the full scope of therapeutic conversation. In one study, 95% of patients experienced being in therapy for at least one problem not covered by the outcome measure being used (Sales et al., 2018). The concern is that a patient experiencing significant trauma symptoms, for example, may feel that these symptoms are not relevant or important if the therapist uses only a brief depression measure to monitor treatment. This problem would be amplified if the therapist de-prioritizes trauma symptoms simply because they are not monitored. This example is not merely theoretical. A systematic review and qualitative meta-analysis of patient perspectives on ROM and feedback reported, as one of four themes, that patients needed measures to capture the complexity of their situation, and felt frustrated when they did not (Solstad et al., 2019). Multiple studies have reported that patients want measures to take a broad approach, including issues such as the alliance, family life and social- and work functioning (Callaly & Hallebone, 2001; Moltu et al., 2018). Moreover, from the clinician perspective, studies have reported that they need ROM and feedback to fit the ways they work in their day-to-day practice (Lavik et al., 2020; Moltu et al., 2018), rather than conflict with it. The importance of measures fitting the concerns of the patient and the training and/or practice of the therapist is a point dating back to Bickman et al.’s (2000) early work in this field. Overall, we can see that misfit between the measure, patient, and treatment can lead to negative impacts on treatment processes, though it is worth noting that these negative outcomes tend to be less common than positive impacts.

The impacts of ROM and feedback on therapeutic processes can indeed be constructive, for example by providing an opening to discuss therapeutically important topics. Hovland et al. (2020) found that patients sometimes interpret items on ROM and feedback as invitations to discuss topics that they otherwise might not bringing up, and that the act of monitoring outcomes with self-report data also evokes helpful patient thoughts and feelings. Using structured measurements also influences which topics are focused on within the therapeutic process (Solstad et al., 2021). Of course, the impacts are not always positive, and it may be the case that both positive and negative impacts co-occur when ROM and feedback are added to treatment (Fornells-Ambrojo et al., 2017).

Whether ROM and feedback ends up being an overall constructive or hindering influence on the therapeutic process is probably not a function of the measure per se, but rather, how the measure is used by both therapist and patient. Aiming to understand why ROM and feedback’s effects on outcomes varied between studies, Krägeloh et al. (2015) reviewed the empirical outcomes literature and categorized included studies in groups characterized by how the measures were implemented. They report that the majority of positive effects of ROM and feedback on outcomes can be found in the group characterized by measures being most closely integrated in clinical practice. Put simply, a specific ROM and feedback measure will lead to different outcomes in different clinics depending on how it is used by the stakeholders in the clinical process. This could serve as a humble reminder for researchers studying technological advances in psychotherapy. While measures may be thought of as a causal intervention, this is a simplification. The agents of change in psychotherapy are the participants.

Brooks-Holliday and colleagues (2021) studied dyads in depth, using semi-structured interview and session recordings, to explore which clinical uses of ROM and feedback were associated with good therapy processes. In dyads where they found a consensus that patient data contributed positively, they report that the therapists focused on providing patients with a helpful rationale for using data to improve their collaboration in treatment. Rationales were used, in other words, to meaningfully integrate patient data into the ongoing clinical conversation. Moreover, the authors report that both explicit discussion connecting patients’ scores with therapy skills and strategies, and actively engaging patients in exploring the meaning of their data were connected to helpful use. This study’s findings nicely illustrate how therapists can use routinely collected data to open meaningful conversations in a way that can engage patients in psychotherapy. The key findings show how important it is to let measurement impact the content of sessions, in order to get positive impacts from ROM and feedback.

Låver and colleagues (2023) reported a systematic review and qualitative meta-analysis of studies exploring how therapists and patients use self-report data in treatment processes. This included and went well beyond the traditional uses external to the therapy process (e.g., for assessment and diagnosis). In this review they also found that patient data prompted interactional processes and facilitated conversations, that patient data enhanced self-awareness, and informed intrapersonal reflections (primarily for patients). Moreover, they report that patients often provided strategic responses to questionnaires in a subset of the included studies, for example to preserve the therapy relationship or get access to services, underscoring how the meaning of patient data cannot be fully assessed outside a clinical conversation. The potential for patient data collection to influence interaction is exemplified by a qualitative result reported by Matanov et al. (2021): “*I said, I would just be sitting down and waiting for him to bring the idea and so that I will bring conversation. But with this it helps to remind me and also help me to speak my mind on what I want to tell him*” (p. 8).

Currently, research into how self-reported data from patient can constructively influence therapeutic process is rather limited in volume. However, the available body of research almost uniformly underscores how the process of collecting such data influences both patients and therapists, evokes thoughts, feelings, and insights in patients, gives direction and focus to the conversation in session, and allows patients another way of communicating their needs.

Understanding routinely collecting data as a strategy influencing the participants and the interaction between them yields an important conceptual question and several implications. This suggests that we should understand ROM and feedback as a clinical skill, as suggested by for example Solstad et al. (2021). If ROM and feedback requires skill, we would need to establish, for training purposes, which kind of skill this is. Is it, for example, a technical or procedural skill, similar to the act of finding the correct diagnosis or teaching a mindfulness exercise? If this is the case, therapists could, in principle, be taught what different pieces of patient feedback meant, and the best steps to take onwards. Alternatively, is it a relational process skill requiring the therapist to both stay attuned and present to the patients’ unfolding story while simultaneously reflecting on how the patient data in combination with other features of the treatment relate to their professional knowledge? If this is the case, the central therapist skill would be integrating the various bits of information from several channels of communication involved in ROM and the clinical treatment itself, to flexibly adjust to the patient’s individual needs. The studies presented in this section suggest, in our view, that both interpretations of “skill” are valid: there are concrete and interpersonal skills involved in successful ROM and feedback interventions. Clinicians should understand that using patient feedback can result in both constructive and unhelpful processes, contingent on how therapists and patients understand and use the data.

### Training in the Clinical Skills of Feedback

Based on the indication that ROM and feedback is beneficial to patient outcomes and the cost-effectiveness of mental health care (e.g., De Jong et al., 2021), it is important that this work is translated into clinical practice and training (see De Jong et al., 2023). There are at least two separable key issues: First, which methods of training help therapists use ROM and feedback more effectively: how do we best train more people in its use? Second, how can ROM and feedback inform existing training in psychotherapy: If ROM and feedback make treatment better, can they help make psychotherapy training more effective overall?

Training therapists in using ROM and feedback effectively consists of several components, and ideally a multi-phased approach is used. In the initial phase, therapists need to learn about the basic principles of ROM and feedback, the practical and technical aspects (e.g., how to use specific ROM software), how to interpret and work with the measures that are used for ROM, and how to discuss ROM with patients. In this phase of training, implementation and training often go hand in hand, and as a result, one of the key objectives of training is to improve therapists’ attitudes toward ROM and feedback. Research has found that therapists who were more positive toward using ROM and feedback were more likely to use it actively in treatment (De Jong et al., 2012) and achieved better outcomes (Lutz et al., 2015). This finding has been replicated in at least one other setting (Lutz, Deisenhofer, Lutz et al., 2022a, b). It may be that a therapist who is willing to be actively informed by patient progress will not only foster more fruitful processes with the patient but will also model an approach to learning and growth from which the patient will benefit. Trainings should start by acknowledging and working through any negative attitudes toward ROM and feedback among therapists, and build positive attitudes instead (see also De Jong et al., 2023). In the second phase of training, the focus is on building confidence and skill in working with ROM and feedback and integrate it into a therapists’ general way of working. This type of training often has the form of coaching and monthly or bi-weekly (peer) supervision, in which therapists discuss their own struggles with ROM (e.g., discrepancies between their own impressions and the feedback), patients’ struggles with ROM (e.g., patients not willing to complete the instruments), and complex cases in which ROM and feedback can support treatment decisions (e.g., help identify hampered therapy processes). This phase is essentially based on the idea that working with ROM and feedback is a skill that needs to be trained and maintained, just like other therapeutic skills.

A second key point is that there is evidence that the use of ROM and feedback seems to induce training effects in psychotherapists, effectively making therapists better over time. A naturalistic study of routine outcome monitoring, feedback, and deliberate practice, involving more than 5,000 patients and 150 therapists, showed a small but statistically significant growth in individual clinician effectiveness over time when the agency implemented the use of routine outcome monitoring coupled with ongoing consultation of especially challenging cases and the planful application of feedback (Goldberg et al., 2016). In a separate trial, Brattland et al. (2018) also found evidence that the effect of ROM and feedback increased over time as the training and support for therapists’ use of the system increased. In a meta-analysis, Delgadillo et al. (2022) found that the use of ROM with feedback led to a reduction in the differences between therapists’ effectiveness.

Psychotherapists, like all people, have some stable characteristics that are trait-like, shaped early in life, and which are not easily modified. Other characteristics are state-like, situational, and more easily trainable. Education and training aim to shape trainees’ relational manner and style; their perceptual, conceptual, and thinking skills; and their specific abilities in carrying out particular techniques and interventions (Knox & Hill, 2021). However, few rigorous, prospective studies have been conducted examining whether the therapist dispositions or skills that are most relevant for therapeutic effectiveness change through focused and intensive training. Despite this, emerging studies within the research program known as the Facilitative Interpersonal Skills (FIS) paradigm indicate that interpersonal skills (verbal fluency, emotional expressiveness, persuasiveness, warmth, positive regard, hopefulness, empathy, and capacity to repair the working relationship via a performance-based test) can be enhanced by psychotherapy training (Perlman et al., 2020, 2023). This suggests that that these more specific characteristics can improve with training, so therapist training likely can have positive impacts even if overall evidence for training effects is mixed.

An important feature of ROM and feedback is that it generates live data on therapists’ treatment outcomes, providing them with an opportunity to learn from these outcomes. This can happen during treatment, for example when a patient gets not-on-track, but also at a more aggregated level during supervision, when reflecting on in which types of patients a therapist is successful and in which types of patients it is harder to get good outcomes for someone (De Jong et al., 2023; Miller et al., 2023). This data can be combined with performance-based measurement of professional skills, like in the Facilitative Interpersonal Skills paradigm (Anderson et al., 2016), to identify patient characteristics and intervention skills that are relatively strong and weak for each therapist. Therapists can then target which skills they are missing and practice those specific skills through the use of deliberate practice (e.g., Ericsson & Lehmann, 1996; Miller et al., 2023). It should be noted that a systematic review concluded that other factors beside therapist skill contribute to therapist efficacy, so training in ROM and feedback should be a part of a therapist training program, not the entire program (Heinonen & Nissen-Lie, 2020; Nissen-Lie et al., 2023).

Overall, the use of ROM and feedback in psychotherapy seems to improve with specific training and can also support the other training needs of psychotherapists. This makes it both an important topic of training, and one that can help improve the overall efficacy of treatment in practice. Work in this area should focus on fostering performance challenges as both measurement and training methods, and take an improvement approach, rather than solely a selection approach. This should be a key feature of training programs and implementation strategies going forward.

### Mechanisms and Components of Feedback

Like many complex interventions, there are numerous potential mechanisms for how ROM and feedback systems affect patient outcomes. Better understanding of these mechanisms is necessary to develop more effective ROM and feedback systems in the future. The most prominent is that feedback may focus clinicians’ attention on cases with the most need, and on the specific topics of most concern, at times when their need is greatest. This greater attention comes about through regularly checking ROM and feedback reports, which vividly identify areas of concern using signals or alarms (most notably, not-on-track notifications). While there is some experimental support for the value of these alarm signals in particular (Delgadillo et al., 2018), very few studies have been conducted to actually test whether such attention-grabbing alerts are causal mechanisms. There is also some evidence that they can be demoralizing in some treatment contexts (Errazuriz & Zilcha-Mano, 2018), suggesting that this mechanism, though promising, may not be sufficient to explain the positive benefits of ROM and feedback.

A second mechanism for ROM and feedback effectiveness is that it might helpfully alter clinicians’ outcome expectations. It seems, like many highly trained professions, therapists suffer from inflated beliefs in their own efficacy and over-optimistic beliefs about patients’ prognoses (Herzog et al., 2023; Kaiser et al., 2022). ROM and feedback may help by providing contradictory evidence to therapists, overcoming this positive bias. Of course, even if feedback primarily acts by changing therapists’ overly-positive expectations, therapists must do something different during treatment to affect change in their patients. This suggests that while altered therapist expectations and beliefs are important, they are not the only process involved in ROM and feedback (McAleavey & Moltu, 2023).

A third mechanism is that ROM with feedback provides new information to clinicians, which would not be available otherwise. This new information may prompt therapists to make changes to treatment, even subtle changes, that better target the patient’s concerns. There is evidence that feedback systems do change the content of therapy sessions. For instance, Douglas et al. (2015) found that when feedback was used, psychotherapy dyads discussed topics that patients thought were important earlier and longer than when ROM and feedback was not used. This may also be true of ROM and feedback systems that include a wider variety of scales than just symptoms and distress. Meta-analytic findings suggest that systems that incorporate additional non-symptomatic data (e.g., alliance, patient motivation) the impact of feedback is greater for patients who are not-on-track (De Jong et al., 2021). Again, this may be a key process in the feedback causal change, but is not itself sufficient: therapists must still use clinical skills with tact and care in order to make use of any new information the measurements uncover.

Finally, there also seems to be evidence that ROM with feedback enhances communication between clinician and patient. This new communication may be a refocusing of the content of sessions on the most important topic, for instance if the ROM feedback suggests that a therapist should inquire about worsening symptoms of depression when they otherwise would not have known to do so (Douglas et al., 2015). It may also be a reframing, allowing for the discussion of patient experiences within a larger or more appropriate context (for instance, rather than discussing a problem experienced in the past week in isolation, ROM and feedback may encourage the patient and therapist to consider it in the context of the entire treatment or larger experience). There is also evidence that ROM causes patients to reflect on their own experiences, which makes it easier for them to discuss these issues in treatment (Solstad et al., 2019). This mechanism may be related to the just-described mechanisms – if therapists pay more attention, have more accurate expectations, and learn new information, their patients may well feel that communication is better – or it may be a fully independent cause of the observed positive effects.

We do not believe these four mechanistic paths – focusing clinicians’ attention on cases with greatest needs, altering clinicians’ outcome expectations, providing new information to clinicians, and enhancing dyadic communication – are the exact or an exhaustive list of ROM and feedback’s effects. Other mechanisms may well be discovered in the future. But they are potential mechanisms which already have some empirical support, and each points to ways to improve the use of ROM and feedback in practice. We also note that these mechanisms are not mutually exclusive: stronger effects from ROM and feedback studies may likely emerge when more than one is activated, and they can all mutually reinforce each other.

## A View Forward

From our current perch at the peak of recent empirical and mechanistic exploration of ROM and feedback, it is possible to provide a few key suggestions for how we should proceed going forward. These are collective pieces of advice and reminders for us all – therapists and researchers alike.

### Look Beyond the Effect of Feedback on Outcome, Especially on the Measure Used for Feedback

It is tempting each time a ROM and feedback system is implemented, to conduct a straightforward effectiveness study comparing past cohorts to a new cohort of patients on the outcome measure used as feedback. While there is nothing wrong with this – and we would encourage the systematic analysis of routine data – the field only needs to invent a wheel once. There is little need for more straightforward two-group studies (especially nonrandomized ones) on whether or not feedback systems “work.” What we do need are more sequential, within-person, and dismantling designs in this field. If we want to better explore mechanisms and look at moderators that make feedback particularly useful or hindering, even well-conducted two-group RCTs will not be enough.

### Feedback Exists in Contexts

Much of the research on ROM and feedback ignores essential cross-cultural and cross-setting differences that make psychotherapy distinct. Whether the patient is being seen in privately-funded long-term outpatient care or intensive inpatient state-funded care will color every other aspect of their treatment. We should not expect effects to be identical across such diverse settings, because treatments are not identical. Treatments themselves are important contextual features, and they will dictate how important different mechanisms can be: a highly emotionally-charged relational or gestalt therapy may require and benefit from enhanced patient-therapist communication, while a more skill-focused CBT may benefit more from new information reaching the therapist, for instance. While certain features of ROM and feedback seem to be consistent across international settings, there are some aspects of feedback that may be considerably different in different regions. For instance, De Jong et al. (2021) found larger effects in American samples than other countries, and Sun et al. (2021) discuss several ways their Chinese sample might respond differently to symptom-focused feedback questionnaires than Western samples. Not only should we expect some differences across contexts, but these differences should be central to how we understand, explain, and use ROM and feedback systems.

### Patient Characteristics

Similarly, the patient is an important contextual factor, even if most of the hypothesized mechanisms of action also require the therapist. We need to do a better job of understanding patient variables that make them more or less amenable to using measures of particular kinds, and whether these are different from the factors that predispose some patients to having good outcomes in psychotherapy without measurement. A considerable portion of the variation in outcomes from psychotherapy is due to patient variables – probably an order of magnitude more than therapist-related variables. Meta-analyses have revealed only a small number of patient-level moderators of feedback effects (De Jong et al., 2021), but the qualitative literature demonstrates considerable variability in how patients experience and use outcome measures (Låver et al., 2023). We must understand this variability to target patients who will benefit most.

### Ensuring That Feedback Contributes to Health Equity

One important reason to investigate patient variables more seriously is that they may help the field ensure that ROM and feedback contribute to increasing health equity. By better understanding which patients require which measurements and interventions, the field can allow better service to historically underserved and lower-resourced individuals who may not have the resources or healthcare system knowledge to advocate for optimal care. ROM and feedback seems to improve the effectiveness of the least effective therapists (Delgadillo et al., 2022), suggesting that it contributes to health equity. However, broad and effective use of ROM and feedback will not happen by chance, and likely requires non-market-based incentives to achieve broad deployment. Governments and research funders must ensure everyone has their needs met. Researchers, however, need to provide information that enables these policies. Data from ROM is perfectly suited to help direct limited resources to patients in most need. By using data from routine care, policy makers can better allocate treatment, provide rapid and more accurate assessments, and monitor cases for emerging needs. Again, this likely will not happen without research that demonstrates feasibility and policies that support these goals.

### ROM and Feedback as a Part of Data-informed Psychological Therapy

ROM and feedback systems are one aspect of what has been recently-termed data-informed psychological therapy (Lutz et al., 2022). This encompasses ROM and feedback as well as more complex interventions including real-time video analysis, computerized treatment recommendations, and automated personalized treatment planning. In this context, ROM data is the linchpin that enables further advances. Patient self-report data from routine data collection is often used to validate new technologically-driven advances in mental healthcare (e.g., Delgadillo et al., 2022), since it is relatively easily available in large samples. This means that researchers interested in feedback should broaden their horizons to include a wider array of data and computer-informed advances in psychotherapy. For instance, advanced research institutes are already capable of linking videos of psychotherapy sessions, extra-sessional activity tracking, sound recording from mobile telephones, and idiographic networks from ecological momentary assessment data with ROM and feedback reports (e.g., Lutz et al., 2019). Psychotherapy will likely persist as a face-to-face format for some time, but eventually, computers will be essential to diagnosis, treatment planning, treatment modification, utterance crafting, and will become more effective at reducing symptoms than human therapists. The systems in use and being built right now will increasingly digitize the entire field. If psychotherapy continues at all in 50 years, it will continue with more computer involvement. Self-report data will retain some relevance, because it remains one of the best ways to measure phenomenologically important processes. But the systems for gaining this data that are popular now, which only ask a handful of simple standard items, are likely to change. Better methods of assessment are sure to come about.

The field should recognize that ROM and feedback are part of the increasingly computerized process of psychotherapy, and each step of computerization alters the therapy process from the moment the patient is registered with a clinic. This provides opportunities to standardize and routinize many psychotherapy processes, building clinical support tools based on what has worked in this field already. However, that does not mean that the future of psychotherapy is monolithic, simple, or inhuman. Rather, the parts of psychotherapy that are often most impactful are related to unexpected or deeply meaningful human contact. Data from digitization can help therapists guide patients to these experiences sooner rather than later. For instance, since we think one of the key mechanisms of ROM and feedback is that it may enhance patient-therapist communication, we should be building systems that are not only highly predictive or cost-effective in trials but building them in ways that further enhance the person-to-person impact of psychotherapy.

One note of caution related to this is that how we use ROM and feedback data is not as simple as it might seem. Data from questionnaires is inherently limited by participants’ biases, understanding, and recollections, even if it is better than many other forms of data for some purposes. Moreover, data from ROM is sometimes provided in contexts with clear and problematic consequences for high or low scores, such as loss of services or changes in compensation. When these additional influences affect score validity (Truijens et al., 2021), using ROM and feedback data as the base of other technology is inherently problematic (Låver et al., 2023).

### ROM, Feedback, and the Further Digitalization of Our Lives

Finally, we envision a not-too-distant future in which computers and artificial intelligence programs are much more heavily involved in psychological therapies (and our lives) than imaginable at present. The field’s duty in an increasingly digital world is to find the tasks for which humans are essential. Most psychotherapists seek to promote some essentially human traits they see in their patients, such as capacity for kindness, resilience, or empathy. Many psychotherapists similarly value the intimate person-to-person contact that is common in psychotherapy. We should consider whether computerized advances help patients and therapists reach those goals, or whether they reduce the chance for human expression and interaction. The advances in statistics, standardized measurements, and clinical feedback systems cannot be useful if they reduce autonomy and capacity for interpersonal contact of psychotherapy participants. In other words, we need ROM and feedback and other data-driven tools to enhance humanity, not just as robotic replacements for biased reasoning. Balancing our needs as human beings with the clear benefits of data-driven decision-making may well be the key challenge in the field.

## Conclusions

ROM and feedback are complex interventions with many areas of uncertainty and growth. These interventions are a major advance to psychotherapy: very few adjunctive interventions have so robustly improved outcomes of diverse psychotherapies. Some of the key areas to further extend this benefit include by advancing personalized and dynamic systems, more effective patient-therapist matching, contextualizing ROM and feedback within specific treatment processes, and improving training in psychotherapy. We believe that a more thorough understanding of the complex mechanistic paths and treatment implications of ROM and feedback will allow for greater uptake and impact on our patients’ lives.
